# Marine Benthic Community Assembly Is Taxonomically Stochastic but Functionally Deterministic in a Dynamic Coastal Sea

**DOI:** 10.1111/ele.70228

**Published:** 2025-10-02

**Authors:** Milenka Sloots, Oscar Franken, Kasper J. Meijer, Tjisse van der Heide, Laura L. Govers, Han Olff

**Affiliations:** ^1^ Conservation Ecology Group, Groningen Institute for Evolutionary Life Sciences University of Groningen Groningen the Netherlands; ^2^ Department of Coastal Systems Royal Netherlands Institute of Sea Research (NIOZ) Den Hoorn the Netherlands

**Keywords:** disturbance, functional traits, macrozoobenthos, stochastic processes, Wadden Sea

## Abstract

Understanding the balance between deterministic and stochastic processes in community assembly is crucial for interpreting ecological community dynamics. Moreover, it provides perspective for conservation and management actions, as deterministic processes can be subject to targeted interventions, but stochastic processes are less manageable. Through a spatially explicit macrozoobenthic monitoring campaign consisting of 1323 sampling locations in the Dutch Wadden Sea, we examined the relative importance of deterministic and stochastic processes, including the role of hydrodynamic disturbance gradients. We found species‐based community assembly to be mainly driven by stochastic processes, while trait‐based assembly was more deterministic and environmentally driven. Environmental disturbance levels minimally affected the relative importance of stochastic and deterministic processes. For coastal benthic ecosystems, we therefore recommend management actions to target specific desired functional groups rather than specific changes in community composition.

## Graphical abstract

We assessed the balance between deterministic and stochastic processes in macrozoobenthic community assembly across 1323 sites in the Dutch Wadden Sea. Species‐based patterns were largely stochastic, while trait‐based patterns were more environmentally driven, with little influence from hydrodynamic disturbance. This suggests that management and monitoring of coastal benthic ecosystems should focus on desired functional traits rather than species composition.

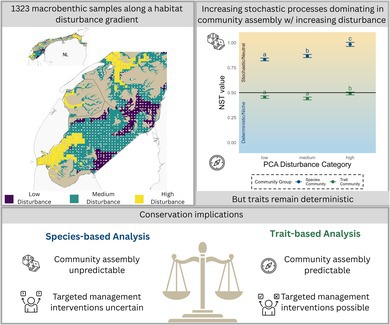

## 1 Introduction

Recognising the main processes that shape the composition of ecological communities can improve predictions of responses to environmental change and help guide management interventions (Chase and Myers 2011; Zhou et al. 2014). Historically, there have been two dominant frameworks for understanding community assembly. Classic niche theory explains the assembly of local communities from a regional species pool based on dispersal ability, environmental filtering and species interactions (Tilman 1982). In contrast, more recent neutral theory assumes functional equivalence of all species, with local community composition driven by stochastic processes such as random birth–death events (ecological drift) and probabilistic dispersal (Chase and Myers 2011; Gravel et al. 2006; Hubbell 2001; Thompson and Townsend 2006), frequently intended as a null hypothesis for patterns of community assembly.

While these theories emphasise different mechanisms, the deterministic–stochastic divide is increasingly seen as an oversimplification. Some community assembly processes, like dispersal, are difficult to classify as stochastic or deterministic (Vellend et al. 2014), and ‘stochasticity’ itself can refer to anything from demographic and environmental variation to measurement error (Shoemaker et al. 2020). Moreover, some argue that stochastic processes produce predictable emergent patterns at larger scales (Shoemaker et al. 2020; Maurer and McGill 2004), or that apparent stochasticity may just be a failure to identify underlying environmental determinants. Nevertheless, certain processes or features of community assembly can be inherently unpredictable (Vellend et al. 2014). Therefore, in this study, we use “stochastic” to refer to community assembly processes that are unpredictable with respect to the identity or characteristics of community members, and ‘deterministic’ to refer to predictable responses to environmental conditions.

Assessing the role of stochastic processes is not only important for a fundamental understanding of ecosystem functioning but also offers valuable insights for conservation and management interventions. Yet, few community assembly studies place their findings in a conservation context (Clark 2009). This is surprising, as conservation managers typically intervene in biotic (e.g., invasive species removal) or abiotic conditions (e.g., reducing eutrophication) to facilitate settlement, persistence, growth or reproduction of key species. Such conservation interventions therefore implicitly assume deterministic community assembly (e.g., Gaston 2000; Hooper et al. 2005; Watson et al. 2014) but often without testing this explicitly. If biodiversity in an ecosystem is instead dominated by stochastic processes, different interventions may be required; for example, with more emphasis on ecosystem size and connectivity (Adler et al. 2007; Economo 2011).

**FIGURE 1:**
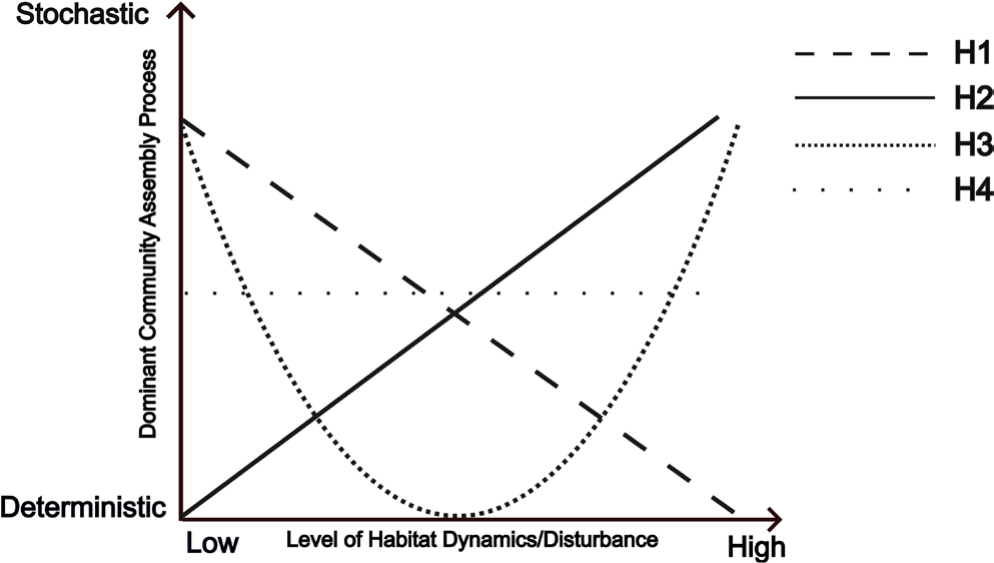
Hypotheses describing the effect of increasing disturbance on community assembly processes. H1: Increasing disturbance leads to more deterministic community assembly (e.g., Chase 2007). H2: Increasing levels of disturbance lead to increasing stochasticity in community assembly (e.g., Ferrenberg et al. 2013). H3: Determinism is highest at intermediate levels of disturbance (e.g., Lepori and Malmqvist 2009). H4: Disturbance levels have no effect on the extent of stochasticity in community assembly.

Unfortunately, there is poor consensus on the main determinants of the position of ecosystems on the deterministic–stochastic spectrum. Different hypotheses exist, especially regarding the role of disturbances such as droughts, fires and flooding (Chase 2007; Ferrenberg et al. 2013; Lepori and Malmqvist 2009). Some studies suggest dominance of deterministic processes in high‐disturbance environments versus dominance of stochastic processes under stable conditions (Figure 1, H1) (Chase 2007). This could be caused by a stronger selection of species that can persist in disturbed environments compared to stable conditions, leading to a strong relation to the environmental conditions. But other studies (primarily focusing on microbial systems) suggest the opposite: dominance of stochastic processes under higher disturbance levels (Figure 1, H2) (Dini‐Andreote et al. 2018; Ferrenberg et al. 2013). In this case, disturbance may randomly eliminate individuals regardless of their fitness, or promote frequent recolonisation from mass effects (Shmida and Wilson 1985). Deterministic processes have also been suggested to become more dominant under intermediate disturbance (Figure 1, H3) (Lepori and Malmqvist 2009). In this scenario, many species can persist in stable environments, leading to weaker relations with the environment, while in highly disturbed areas, random birth/death events and mass effects drive community assembly (Lepori and Malmqvist 2009; Shmida and Wilson 1985). Finally, the importance of stochastic and deterministic processes might be generally independent of disturbance (Figure 1, H4).

The lack of consensus is exacerbated by the fact that most studies related to stochastic community assembly methods have focused on terrestrial ecosystems or microbial communities (e.g., Conradi et al. 2017; Ferrenberg et al. 2013; Mori et al. 2015; Shipley et al. 2012). Aquatic and benthic environments, especially marine environments, differ strongly in certain ecological and evolutionary processes compared to terrestrial systems (Carr et al. 2003; Gagné et al. 2020). Yet community assembly processes in these environments remain poorly studied (Heino et al. 2015). Physical and chemical gradients are generally steeper in marine environments (Giller et al. 2004), and marine diversity is typically less well explained by environmental variables than terrestrial diversity (Gagné et al. 2020). In addition, mobility and connectivity are generally higher in marine compared to terrestrial environments—for example, due to pelagic life stages (Armonies and Reise 2003; Byers and Pringle 2006; Carr et al. 2003; Giller et al. 2004), although dispersal rates strongly differ between marine species (Grantham et al. 2003; Kinlan and Gaines 2003). Species groups with limited dispersal ability may be more affected by ecological drift, as population isolation and reduced colonisation ability increase a population's susceptibility to random events (Evans et al. 2017; Powell et al. 2015; Pringle and Wares 2007; Stegen et al. 2015). On the other hand, source‐sink dynamics can allow the persistence of well‐dispersed species in areas with unfavourable conditions due to a high supply of individuals from source populations, weakening the perceived species–environment relationship (Leibold et al. 2004; Stegen et al. 2015). These aspects may strongly impact community assembly processes and therefore marine communities may be assembled very differently from terrestrial communities (Giller et al. 2004; Kinlan and Gaines 2003). As conventional management strategies target deterministic community assembly processes, marine communities may need a different set of management interventions aimed at protecting biodiversity.

To position marine communities on the deterministic‐stochastic spectrum in a dynamic ecosystem, we analyse data from a large monitoring program of macrozoobenthic communities in the Dutch Wadden Sea sampled in a grid with a kilometre‐scale resolution. Besides taxonomic community composition, we also assess the community composition from a functional trait perspective. Similar species within functional groups are typically assumed to be more functionally equivalent (making neutral processes more important), while between functional groups, niche differences are expected to be larger. A trait‐based approach may therefore be more suitable for understanding the impact of environmental conditions on species assemblages (Bremner 2008). Furthermore, the Wadden Sea is particularly suitable to test the effects of disturbance on the relative contribution of community assembly processes, as this shallow sea is characterised by strong gradients in hydrodynamic conditions, due to variation in currents, tides and waves (Meijer, Franken, et al. 2023; Reise et al. 2010). We therefore also investigate the impact of the disturbance intensity on the position of communities on the deterministic‐stochastic spectrum (Figure 1). Based on the results, we highlight consequences for conservation and management.

## 2 Materials and Methods

The Wadden Sea is the world's largest interconnected intertidal soft‐bottom ecosystem, spanning three countries. It is a highly productive ecosystem with high biodiversity and crucial ecological functions, including providing a stopover habitat for migratory birds and key nursery and breeding grounds for various fish and bird species (Reise et al. 2010). This unique ecosystem consists of barrier islands, intertidal flats, salt marshes, shallow subtidal flats and deeper tidal gullies and channels (Philippart and Epping 2010). The morphology of the system is very dynamic due to the constant action of waves, tides and currents, as well as ongoing erosion and deposition of sediment (Wang et al. 2012). In this study, we use the subtidal (permanently submerged) Dutch part of the Wadden Sea as our model ecosystem. In the subtidal, sandy sediments show a gradient from coarse grained at the tidal inlets to fine sediments near tidal devices and coastlines (Colina Alonso et al. 2022), which undergo constant redeposition (Ricklefs et al. 2022). The subtidal Wadden Sea also experiences strong gradients in local hydrodynamic conditions and salinity (Philippart and Epping 2010).

### 2.1 Species Community Data

Benthic macrofauna data was collected in a large‐scale sampling campaign in 2019, which sampled 1323 sites. Of these sites, 985 were spatially distributed in a grid with 1 km spacing between adjacent sampling sites, and 338 sites were randomly positioned in‐between these grid points (Appendix S1, Franken et al. 2025). These locations were added to be able to make semivariograms that include distances below the 1 km scale, which ensures that we do not miss critical small‐scale effects of spatial autocorrelation. The research vessel RV Navicula was used to sample 765 deeper sites using a ~20 × 30 cm boxcore, taking samples of approximately 20 cm deep. At 558 shallower sites, which the research vessel could not reach due to its draught, four 10.4 cm diameter manual cores, also with a depth of ~20 cm, were taken from a rigid inflatable boat and pooled. This was a practical sampling limitation, as it was not possible to use the larger, heavy boxcore from the rubber boats. Also, crew and boat time resources did not allow a doubling of the sampling design (from 4 to 8 manual cores) to sample the same total surface area per location with manual cores compared to the box cores. While the total surface area of the four pooled manual cores (346 cm^2^) per sampling location was therefore smaller than the boxcore surface area (639 cm^2^) per sampling location, the pooled manual core samples were taken slightly apart from each other across an approximately 2 m^2^ area. This larger sampled extent partly compensates for the smaller total area sampled, but as the samples were taken underwater in turbid water, the exact extent was unknown, making it impossible to define a clear correction factor. Because of this, species richness may be underestimated in shallow sites since a smaller area was searched. All species data was therefore expressed as abundance per square meter for each sampling location by multiplying the number of individuals by 1/0.0346 = 28.9 for the pooled manual core samples, and 1/0.0639 = 15.6 for the single box core samples.

All samples were sieved over a 1 mm mesh. Larger shellfish were frozen for transportation and storage, while the remaining fauna were preserved in a pH‐buffered 6% formaldehyde solution. For sorting, these samples were stained with Rose Bengal (CAS Number: 4159‐77‐7). In the lab, each individual was identified to the lowest taxonomic level possible (usually species). For each taxon, the abundance was standardised to a density per square metre. The density data was Hellinger‐transformed to mitigate the effects of overabundant species. As there were 22 samples in which no biota was found, a dummy species with a (Hellinger transformed) density of 0.01 was added to all sites in order to retain the 22 sampling locations in the analysis and link the position to abiotic data. After processing and accounting for sites that have both biotic and abiotic data, 1252 sampling locations and 148 taxa (of which 120 identified to the species level) plus 1 dummy species remained and were used for further analysis.

### 2.2 Abiotic Data

We assembled 24 spatial layers of abiotic data for our study area, including the bathymetry, sediment characteristics, salinity and hydrodynamic metrics. These variables were obtained from various sources: Sediment characteristics were interpolated from our field surveys, while others were produced by governmental agencies (bathymetry related) or other research institutes (hydrodynamic metrics, van Weerdenburg and Vroom 2021). All abiotic layers, the reference period used for their calculations, and their sources are described in detail in Appendix S2. We standardised each abiotic variable by scaling to zero mean and unit variance and checked for multicollinearity using Variable Inflation Factors (VIF); removing variables with a VIF higher than 5 (Alin 2010) using stepwise removal based on Pearson pairwise correlations. Twelve abiotic variables were retained for use in variance partitioning analyses: Terrain Ruggedness Index (TRI), standard deviation of bathymetric turnover, silt percentage, median grain size, standard deviation of salinity, maximum salinity, mean orbital velocity, bed shear stress, slope of seafloor and Bathymetric Positioning Indices at kernel distances of 1 km, 5 km and 15 km (see Appendix S3 for map overviews).

To create a proxy for habitat disturbance, we preselected the standardised abiotic variables that were related to currents, waves or sediment movement (11 out of 24). These were then separately checked for collinearity, removing all variables with a VIF higher than 5. From this step, seven variables were retained: Mean orbital velocity, median grain size, bed shear stress, standard deviation of bathymetric turnover, TRI, slope of seafloor and silt percentage. We conducted a Principal Component Analysis (PCA) on these variables. The first principal component (PC1) was strongly associated with the disturbance variables, and an equal number of sites were classified as either low, medium or high disturbance based on this PC1 score (see Appendix S4 for abiotic variable distribution across disturbance categories). For visualisation, we also interpolated the PC1 values at each site to a 1 km by 1 km raster using Inverse Distance Weighted Interpolation in QGIS 3.34 with a distance coefficient of 2.

### 2.3 Trait Data

To test whether the level of stochasticity depended on the use of taxonomic (species data) versus functional (trait‐based) communities, we used a functional trait community as an alternative to the species community. For this trait‐based community, we used data from a macrozoobenthic species database for the Dutch Wadden Sea (Bosco Gusmao et al. 2022; Meijer, Gusmao, et al. 2023). This database includes information on 235 taxonomic groups and their life history traits (such as adult movement type, fecundity and larval development location), divided into specific categories called modalities. For instance, the life history trait describing adult movement has four modalities: sessile, swim/float, crawl/walk and burrow/tube. A complete list of the life history traits used is described in Appendix S5. Using a fuzzy‐coding method (Chevene et al. 1994), each modality is scored between 0 and 3. A score of 0 means no affinity with that modality, while a score of 3 indicates exclusive affinity. Taxa exhibiting affinity to multiple modalities receive scores of 1 or 2, with 1 indicating a weaker affinity and 2 indicating a stronger or equal affinity. We created the trait‐based community dataset by multiplying the abundance of each species with the modality score of 16 trait categories. Species abundance*modality scores were then summed over each site and divided by the total abundance per site to calculate the community‐weighted mean.

### 2.4 Spatial Data

To assess to what extent variation in community composition could be attributed to spatial structure between the 1252 sites, we created distance‐based Moran's Eigenvector Maps (db‐MEM), using the ‘adespatial’ package in R (Borcard and Legendre 2002; Dray et al. 2006, 2023). A Euclidean distance matrix was constructed using site coordinates, then truncated so that only the distances between close neighbours are kept. The truncation threshold was set as the maximum point‐to‐point distance in the minimum spanning tree connecting all sites (2270 m); distances beyond this threshold were assigned an arbitrarily large distance value, in this case, four times the threshold value. On the truncated distance matrix, we computed a Principal Coordinate Analysis (PCoA) and the eigenvectors that maximise Moran's autocorrelation index were extracted (the db‐MEMs). To select the MEMs most relevant to this study, we conducted a Redundancy Analysis (RDA) on the community data using the MEM vectors as explanatory variables (Dray et al. 2006), then used forward selection to retain only the MEMs that increased adjusted *R*‐squared of the RDA, discarding the rest. Thus, we ensured that any superfluous MEM vectors that did not contribute to the spatial explanatory power of the community data were discarded.

### 2.5 Data Analysis

We used three different methods to investigate community assembly processes across a spectrum of habitat dynamics: Detrended Correspondence Analysis (DCA), variance partitioning and a null model analysis.

#### 2.5.1 Detrended Correspondence Analysis

DCA was used for an initial identification of potential patterns in variation in community composition. We performed a DCA on both the Species × Site matrix and Trait × Site matrix, using the *vegan* package in R (Oksanen et al. 2024). PC1 and PC2 components from the PCA that were conducted on the abiotic variables were then plotted over the DCA to identify correlations between natural disturbance and patterns in community composition.

#### 2.5.2 Variance Partitioning

Variance partitioning was used to partition the explained variance in community composition into spatial, environmental or spatially structured environmental processes (Borcard and Legendre 2002; Chase and Myers 2011; Legendre et al. 2005). Communities with a substantial variance attributed to pure spatial processes can be interpreted as more neutrally assembled. A large proportion of residual variance can either indicate missing explanatory variables or a strong influence of random stochastic events (Chase and Myers 2011; Cottenie 2005; Legendre et al. 2009; Leibold et al. 2004).

Variation partitioning was performed in R using the ‘*varpart*’ function from the ‘vegan’ package (Oksanen et al. 2024). Spatial and Environmental Variables were separated into two matrices, and a variation partitioning of the community data as explained by the two matrices was conducted using Canonical Correspondence Analysis (CCA). An adjusted *R*‐squared value for each variance partition was calculated, and *p*‐values obtained through permutation for each pure partition (i.e., excluding the variation explained by both spatial and environmental variables). Variation partitioning was carried out for both the Species × Site matrix and Trait × Site matrix.

#### 2.5.3 Null Model Analysis

We also conducted a null model analysis to quantify the relative influence of stochastic versus deterministic processes on community composition (Chase and Myers 2011; Tucker et al. 2016), using the Normalised Stochasticity Ratio (NST), which compares observed pairwise community dissimilarities to those generated under a null model (Ning et al. 2019). To do so, the abundance‐weighted Jaccard (dis)similarity was used. The null model was run on the regional species pool, where each community is composed of drawing species from the regional species pool. The regional species pool in this study was defined as all the species/trait modalities used in that particular analysis (e.g., for analysis on the low dispersal category, all species within this category were used as the regional pool). Here, the number of species per site was fixed based on the number of observed species in that location to keep alpha diversity representable for those locations. The probability of drawing a species was proportional to its observed occurrence frequency in the respective (species or trait‐based) dataset. The null model was run 1000 times for each site. The approach gives an NST value which ranges from 0 (fully deterministic community) to 1 (fully stochastic community) for each site, with an NST value of above or below 0.5 indicating a dominance of stochastic or deterministic community assembly processes, respectively. Bootstrapping with 999 random draws was used to evaluate the distribution of NST values within and between each disturbance group (Ning et al. 2019).

## 3 Results

### 3.1 Abiotics and Habitat Disturbance Classes

**FIGURE 2:**
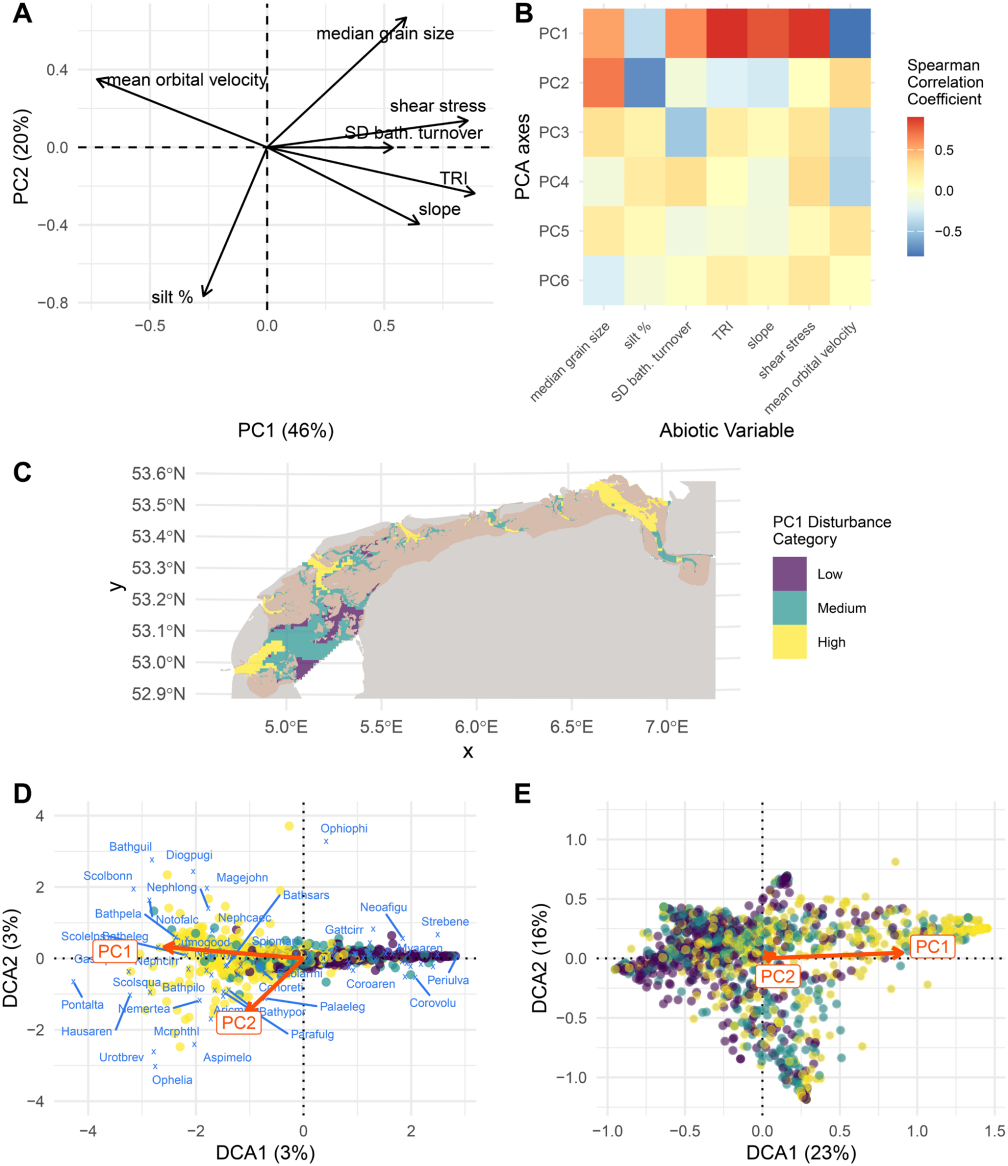
Overview of the abiotic and biotic data used in this study. With (A) the results of a PCA analysis on the disturbance‐related abiotic factors, (B) a correlation heat map of these abiotic factors with the PC axes, coloured according to the Spearman correlation coefficient. Panel (C) shows the categorised PC1 values on a map of the subtidal areas of the Dutch Wadden Sea (purple for Low, teal for Medium and yellow for High disturbance) for the area that was samples. Intertidal areas (beige), land (grey) and different water bodies (white) were not sampled for this study. Panel (D) shows the DCA results for species community data and (E) for the trait community data, including loadings of the PC1 and PC2 axes. In D, species codes are plotted in blue text. In D and E, sites are plotted as points coloured according to the disturbance categories from C.

We first created a proxy for habitat disturbance that could be used in subsequent analyses, using the scores of a principal component analysis on seven disturbance‐related abiotic variables. The first two principal components explained 46% and 20% of the observed variation in disturbance, respectively. The first principal component (PC1) was strongly associated with environmental disturbance factors, with negative PC1 values representing a more wave‐driven, lower disturbance habitat and positive PC1 values representing a higher disturbance and a more current‐driven habitat (Figure 2A,B). The second principal component (PC2) mainly reflected seafloor sediment properties, with high values indicating a higher median grain size and a lower silt content. As PC1 is strongly associated with the disturbance variables, these scores were used for subsequent data analysis. We classified PC1 scores such that an equal number of sites in the study area were classified as either low disturbance (PC1 scores −5.2 to −1.16), medium disturbance (PC1 scores −1.16 to 0.84) or high disturbance (PC1 scores 0.84–5.7) (Figure 2C).

### 3.2 Detrended Correspondence Analysis

To explore environmental gradients shaping species turnover, we used DCA ordinations of both the species and trait communities. Ordination at the species level revealed that little variation in community composition was explained by the first two ordination axes (3% and 3%, Figure 2D). This means that no particular order (scores) of sites could be found that clearly separates species into different niches when abundances are plotted against these site scores. But when the ordinations were done at the level of trait (aggregating species with the same traits in functional groups), the ordination axes explained much more variation in community composition (23% and 16%, Figure 2E). The abiotic disturbance gradient was significantly correlated with the first (PC1) and second (PC2) DCA ordination axes for both species (Figure 2D, and see Appendix S5 for an overview of PC1 values per species) and trait communities (Figure 2E), with much higher adjusted *R*‐squared values for the species community (0.26 for PC1 and 0.18 for PC2) compared to the trait community (0.15 for PC1 and 0.01 for PC2), following a permutation test (*p* < 0.001 for both).

In the trait‐based DCA, high DCA1 values aligned with high PC1 values, so with the more disturbed and rugged seafloor conditions found in the high disturbance category (Figure 2E). The trait modalities clearly separated along the DCA1 axis (Appendix S6). High DCA1 values/PC1 values were correlated with early sexual maturation (≤ 1 year; DCA1 = 0.73), short lifespans (≤ 1 year; DCA1 = 0.70), low offspring numbers (1–50; DCA1 = 0.80), burrowing movement (DCA1 = 0.80) and bioturbation as surficial modification (DCA1 = 0.61). High DCA1/PC1 values were also linked to brooding reproductive modes (DCA1 = 0.67), continuous reproduction (≥ 2× per year; DCA1 = 0.68), benthic/direct development (DCA1 = 0.81) and shallow living depths (surface to 8 cm; 0.48–0.59). In contrast, negative DCA1/PC1 values were linked to planktotrophic larval development (DCA1 = −0.40), broadcast spawning (DCA1 = −0.53), semelparity (DCA1 = −0.49), single annual spawning events (DCA1 = −0.34) and tube‐dwelling species (DCA1 = −0.55). In summary, DCA1 seems to represent a continuum from slow to fast life histories (Jeschke and Kokko 2009), where a fast life history is characterised by early reproduction, short generation time, short lifespan, small adult body size, small offspring size, and high fecundity, and slow ones showing the reverse (Reynolds 2003).

### 3.3 Variance Partitioning

**FIGURE 3:**
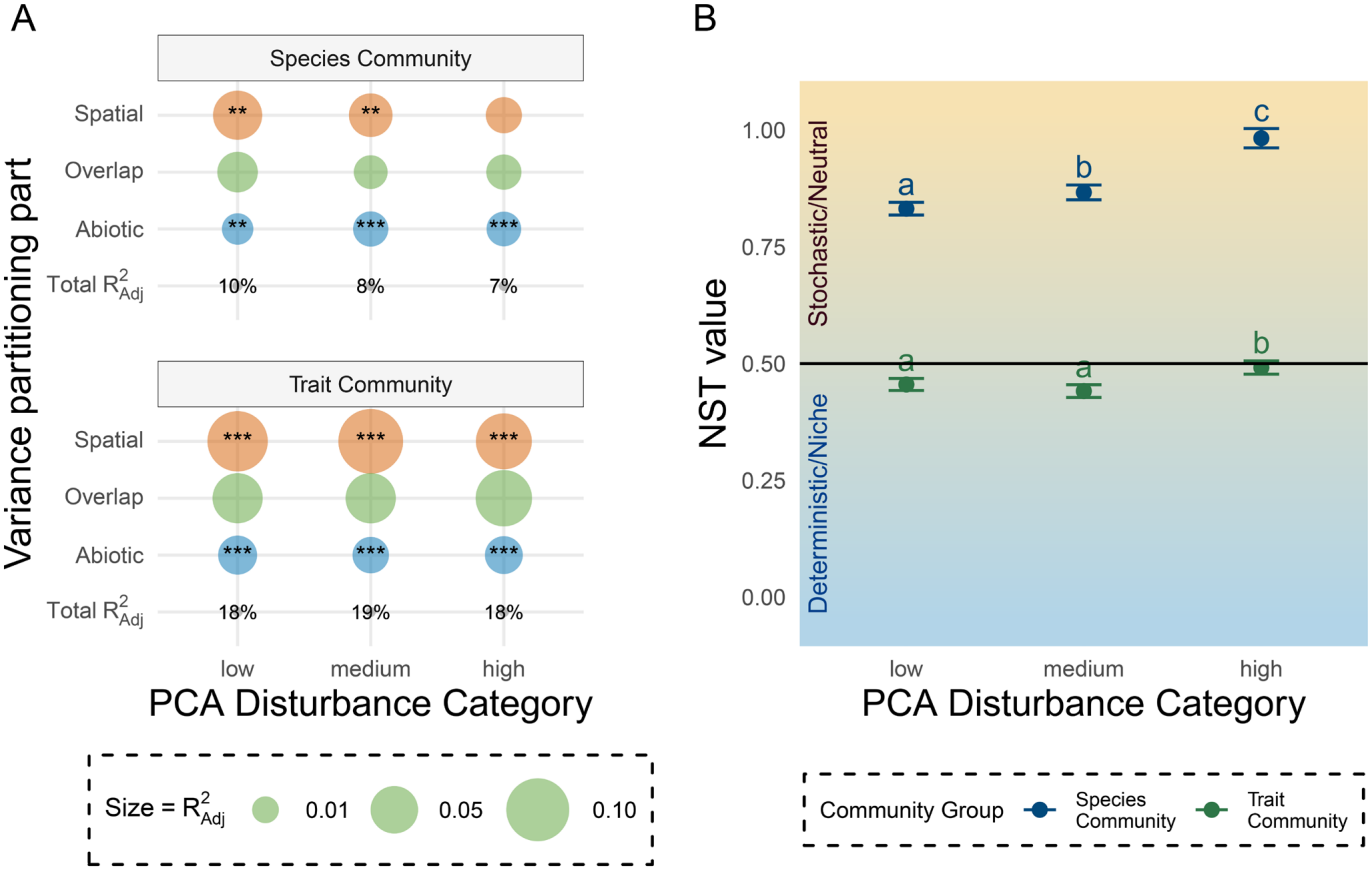
Summary of variance partitioning (A) and null model (B) results, along three categories of disturbance and split between species and trait communities. (A) Explained variance (Adjusted *R*‐squared) is divided into the fraction of variation explained purely by spatial factors (Spatial), purely by abiotic factors (Abiotic) and the shared fraction of variation that cannot be solely attributed to abiotic or spatial factors (Overlap). Stars represent the significance of fractions in explaining community composition. Adjusted *R*‐squared is represented by the size of bubbles, with the total explained variation for each disturbance category converted to a percentage displayed. (B) Normalised Stochasticity Ratio values between 0.5 and 1 indicate a larger role for stochastic processes in community assembly, while NST values between 0 and 0.5 indicate a larger role for deterministic processes. Different letters indicate statistical differences (*p* < 0.05) between means of a species‐ or trait‐based community, not for statistical differences between the different community datasets.

Consistent with the DCA analysis, variance partitioning also revealed a higher total explained variance for trait communities compared to species communities (Figure 3A). Total explained variation ranged between 7% (high disturbance) and 10% (low disturbance) for the species community. By contrast, 18% (high disturbance) to 19% (low disturbance) of the trait community composition could be explained, of which the largest part was explained by spatial variation. For both the trait‐based and species‐based variance partitioning, both abiotic and spatial factors contributed significantly to the total amount of explained variation in community composition. However, for almost all disturbance categories, spatial components contributed more to the explained variation than the abiotic factors. The exception to this pattern is the high disturbance category in the species‐based analysis, where only the abiotic factors contributed significantly.

### 3.4 Null Model Analysis

We conducted a null model analysis to indicate whether community assembly can mainly be explained by deterministic (value < 0.5) or stochastic (value > 0.5) processes. For communities based on the species composition, NST values fell in the stochastic range (0.83 ± 0.014; 0.87 ± 0.016 and 0.98 ± 0.021 for the Low, Medium and High disturbance categories, respectively; Figure 3B). However, when using traits instead of species data, the NST values fell in the deterministic range (0.45 ± 0.013; 0.44 ± 0.014 and 0.49 ± 0.014 for low, medium and high disturbance categories; Figure 3B). Both the species and trait communities had significantly higher NST values in high disturbance areas compared to those in low and medium disturbance areas (bootstrap test, both *p* < 0.05), indicating that stochastic processes play a somewhat larger role in high disturbance areas. Interestingly, the difference between high and the other disturbance categories was relatively small for trait communities (an increase of 4%), but substantial (15%) for species communities (Figure 3B).

## 4 Discussion

In this study, we tested the relative contribution of deterministic versus stochastic community assembly processes over a disturbance gradient in a marine ecosystem, using macrozoobenthic communities in the subtidal Dutch Wadden Sea to test predictions from niche and neutral theories. We found that stochastic (neutral‐based) processes were more dominant compared to deterministic (niche‐based) processes in determining the community assembly when analysed at the species level. However, when the community was analysed at a trait level, deterministic processes increased in relative importance and became more evenly balanced with stochastic processes. Therefore, we found that species‐based community patterns correspond more closely to neutral theory than those based on functional traits. These findings highlight the importance of trait‐based approaches for the monitoring and management of marine ecosystems, as they may provide more consistent signals of community responses to environmental change in dynamic systems like the Wadden Sea.

The results of our three methods of ordination, variance partitioning analysis and the null model analyses all suggest that stochastic processes were highly important in the community assembly of macrozoobenthic communities at the species level, thus aligning more with the assumptions of neutral theory. This matches with the general idea that marine benthic ecosystems are strongly influenced by stochastic processes due to generally high connectivity combined with high disturbance, reducing the influence of environmental gradients (Cottenie 2005; Heino et al. 2015). Other studies support this: for example, a study on intertidal macrozoobenthic communities in the Dutch Wadden Sea reported an environmental explained variance of only 4%–9% (Compton et al. 2013); and a meta‐analysis found that the majority of macroinvertebrate communities in marine and estuary ecosystems exhibited strong spatial structuring unrelated to environmental variables (Cottenie 2005). However, more sheltered and stable coastal environments, such as the northern Baltic Sea and Senegalese mangroves, showed higher explained variation in species composition (44%–46% and 25%, respectively; (Jacquot et al. 2023, 2024)). These differences in the importance of stochastic processes between ecosystems may therefore be determined by differences in their degree of environmental disturbance.

Within the Wadden Sea, we found a significant effect of an increased contribution of stochastic processes under the highest disturbance regime for both the species‐ and the trait‐based null‐model analyses, consistent with hypothesis H2 (Figure 1). However, though the differences between the high disturbance and medium and low disturbance levels were significant, they were very small, which resembles the pattern of hypothesis H4 (i.e., limited effect of the disturbance gradient). This lack of strong effects may be attributed to the overall highly dynamic nature of the Wadden Sea (Rippen et al. 2021; Wang et al. 2012). While our study sites do vary in the level of disturbance, the three disturbance categories have been classified based on the relative differences in abiotic conditions between sites. It might be the case that all our sites experience relatively high disturbance compared to other ecosystems, and that all three categories actually represent high disturbance levels, for example, are positioned on the right side of the disturbance gradient in Figure 1, restricting our ability to distinguish among the H2 and H4 hypotheses. Therefore, to complement our results and to disentangle these two hypotheses, similar analyses could be carried out in coastal ecosystems representing an even wider range of abiotic conditions.

In determining which processes contribute most to community assembly, it is important to acknowledge that the quality of the primary data can affect the degree to which patterns can be statistically recognised. An alternative explanation for high levels of unexplained variation and high contributions of stochastic processes could lie in the quality of the collected data. The sampling campaign used here covers a large total surface area and is therefore expected to capture the large ecological gradients present in this ecosystem (Kraan et al. 2015). However, on the scale of individual samples, spatial sampling resolution, taxonomic resolution of identifications, sampled surface area, different methodologies and sampling depth in the sediment can all affect the extent to which the sampled community represents the actual local community composition (Thrush 1991). In particular, in our study, there can be substantial small‐scale spatial heterogeneity, meaning that samples taken closely together may not contain the exact same macrozoobenthic community. Taking samples over a larger surface area could reduce this degree of ‘sampling stochasticity.’ Likewise, sieving over a coarser mesh size would likely result in a more ‘stable’ (and thus deterministic) community, as the reduction of taxonomic variation in smaller organisms (e.g., polychaetes) would reduce the amount of relatively rare species (Callaway et al. 2002; Wijsman et al. 2022). Such differences in methodology would likely result in the detection of a higher contribution of deterministic processes to community assembly. While it is important to acknowledge the possible differences between sampling techniques and their ability to identify deterministic or stochastic community assembly processes, such an analysis is beyond the scope of this study. It would require a direct comparison between different sampling techniques, which is highly recommended for future studies.

Compared to the species‐level analyses, we find that community assembly processes become much more deterministic when using trait‐based community data. Trait community data were more structured along environmental gradients, as portrayed in the variance explained by the DCA axes. In addition, the trait community deviated more from the null model community, and more variation in community composition was explained in the variance partitioning analysis. This is confirmed by other trait‐based ecological studies, and ecological analyses using a functional perspective are therefore becoming more common (Bremner et al. 2003; Lavorel and Garnier 2002; McGill et al. 2006; Westoby and Wright 2006). For example, variance partitioning of benthic macroinvertebrates in rivers revealed a higher explained variance from environmental variables at the functional level compared to the taxonomic level (Feld and Hering 2007). This suggests that a trait‐based approach is more effective in uncovering the impacts of changes in abiotic conditions on communities. This is likely because environmental filtering processes act on species traits, not on individual species identities. For instance, a certain sediment type might select for burrowing species, regardless of taxonomic identity. By grouping species based on functional traits, trait‐based analyses account for functional equivalence—as described in neutral theory—that often leads to unpredictable community assembly patterns at the species level. This also highlights that the neutral versus niche contrast for ecological communities is overly simplistic; the degree of neutrality depends on how communities are characterised. Therefore, in cases where the dynamics of individual species are highly stochastic, and communities contain many rare species, a trait‐based analysis of abiotic conditions on communities is more robust, and is therefore also more effective in steering and evaluating management interventions in shallow coastal ecosystems such as the Wadden Sea.

## 5 Concluding Remarks

Conventional conservation strategies for coastal ecosystems, such as reducing pollution or disturbance, implicitly assume that the species composition of marine communities is mainly driven by deterministic processes (Myers et al. 2000). This means that changing environmental conditions through appropriate management can alter community composition in a desired direction. But this is less applicable when stochastic processes dominate community assembly and biodiversity. In that case, management could focus on preserving or strengthening regional species pools to maintain stable source populations. The size of marine protected areas is important in that regard, as larger protected areas typically encompass a higher connectivity between suitable habitat patches. This drives overall diversity patterns, lowering extinction rates at the seascape level and preserving gamma diversity (Economo 2011). In addition, we conclude that monitoring and evaluation of conservation interventions can benefit from a trait‐based perspective, as the composition of trait‐based communities is more deterministic than that of species‐based communities. We therefore recommend management actions to target and monitor desired functional groups rather than specific changes in community composition.

## Author Contributions

H.O., M.S., O.F. and K.J.M. conceived the idea; M.S. conducted data analysis, with key contributions from O.F. and K.J.M.; M.S. led the writing of the manuscript; all authors contributed or provided input to draft versions.

## Peer Review

The peer review history for this article is available at https://www.webofscience.com/api/gateway/wos/peer‐review/10.1111/ele.70228.

## Supporting information


**Appendices S1–S6:** ele70228‐sup‐0001‐AppendicesS1‐S6.docx.

## Data Availability

Scripts and data that support the findings of this study are openly available in DataverseNL at https://doi.org/10.34894/L8VE00. Benthos species data will be accessible through https://doi.org/10.25850/nioz/7b.b.qj, macrozoobenthos trait data is available from https://doi.org/10.34894/Z43J6I.
