# Supplementary material for: Marine Benthic Community Assembly Is Taxonomically Stochastic but Functionally Deterministic in a Dynamic Coastal Sea
Source: Ecol Lett. 2025 Oct 2;28(10):e70228. doi: 10.1111/ele.70228 (PMC12490742; doi:10.1111/ele.70228)
Supplement: Supplementary file 1 — Appendices S1–S6: ele70228‐sup‐0001‐AppendicesS1‐S6.docx. [file ELE-28-0-s001.docx]

# Appendix S1: Macroozoobenthic Sampling Points from 2019 Sampling Campaign

This map is adapted from Franken et al., 2025. Points indicate sampling locations used for the study; intertidal mudflats are shown in beige and land is shown in grey. A distinction was made between the Boxcore samples in the deeper locations (dark blue) and the Manual cores in the shallower locations (light blue).


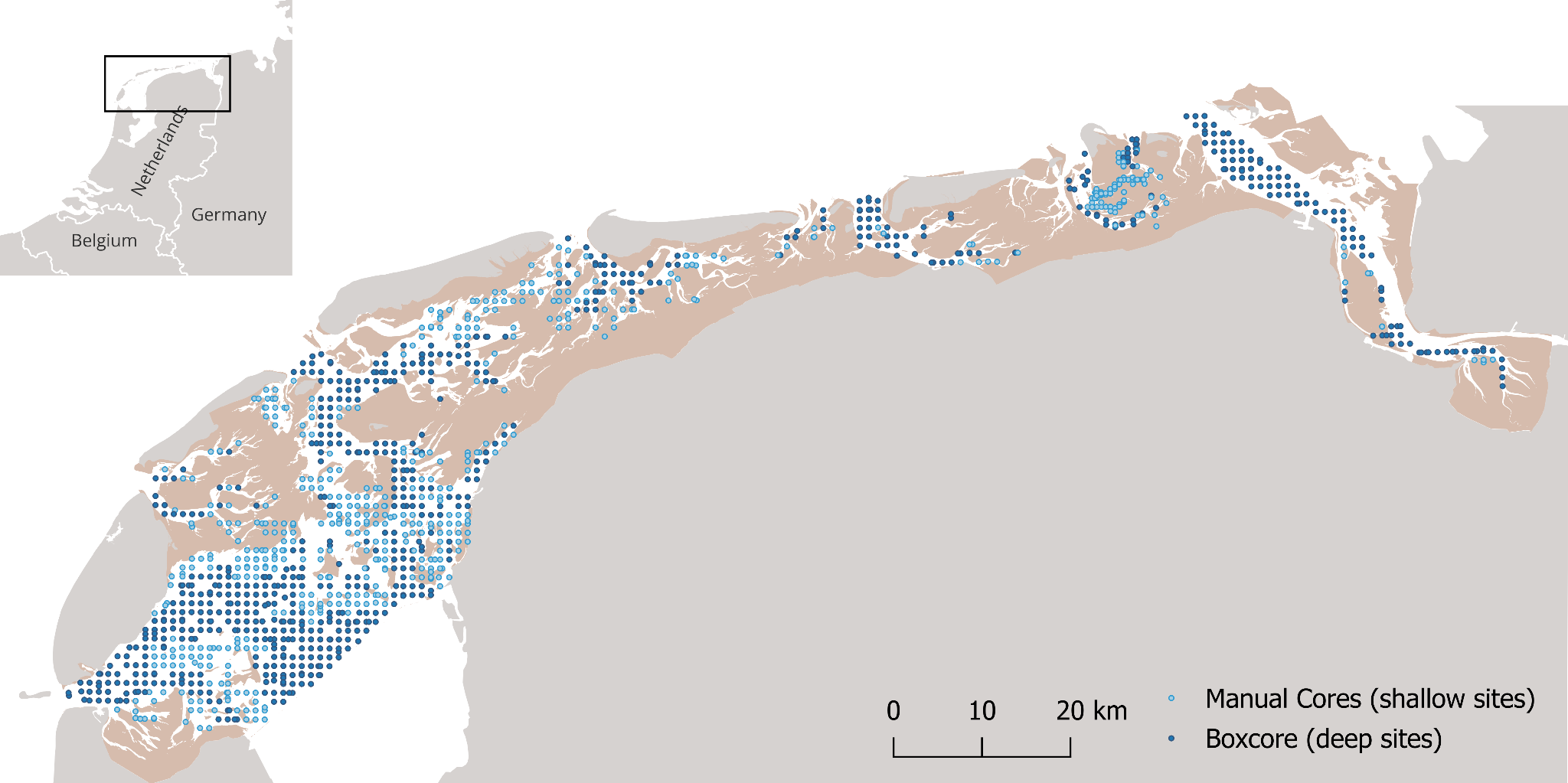


# Appendix S2: Description and Sources of abiotic variables

| **Supplement 1:** Description of each of the abiotic variables considered in this study, including their sources and which stage of the data analysis they were included in (if any) | | | |
| --- | --- | --- | --- |
| **Abiotic Variable** | **Description** | **Source** | **Where used in Analysis?** |
| Bathymetric Positioning Index | Bathymetric Positioning Index (BPI) was calculated using bathymetry data for 2015 - 2020. BPI was calculated using different kernel sizes (1km, 5km, 15km, 25km and 50km). | (Meijer et al. 2023a) | BPI 1km, 5km and 15km were used In the abiotic explanatory dataset |
| Bathymetry | Bathymetric data obtained by Rijkswaterstaat for the period 2015-2020, at 20m resolution. |  | - |
| Shear stress | Modelled using the 2D version of the Dutch Wadden Sea Model made by Deltares, at 100m resolution. Based on two full tidal cycles from 23 June to 22 July 2017, which were representative for the years 2013-2017. | (van Weerdenburg & Vroom 2021) | In abiotic explanatory dataset    PCA analysis |
| Current Velocity | Calculated using the 2D version of the Dutch Wadden Sea Model made by Deltares, at 100m resolution. Based on two full tidal cycles from 23 June to 22 July 2017, which were representative for the years 2013-2017. | (van Weerdenburg & Vroom 2021) | - |
| Orbital Velocity (April 2020) | Calculated for the months January, March and April of 2020, using the SWAN-Kuststrook model, based on wave measurements of the respective months, at 100m resolution. | (van Weerdenburg & Vroom 2021) | - |
| Orbital Velocity (January 2020) |  |  | - |
| Orbital Velocity (March 2020) |  |  | In abiotic explanatory dataset    PCA analysis |
| Mean Salinity | Calculated using the 3D version of the Dutch Wadden Sea Model made by Deltares, at 200m resolution. Based on the yearly average values of 2017. |  | - |
| Maximum Salinity |  |  | In abiotic explanatory dataset |
| Minimum Salinity |  |  | - |
| Standard Deviation in Salinity |  |  | - |
| Variation in Salinity |  |  | In abiotic explanatory dataset |
| Median Grain Size | Obtained from the same large-scale sampling campaign as the species data in 2019. Kriged to interpolate data for the entire Wadden sea, at 200m resolution. | doi 10.25850/nioz/7b.b.qj | In abiotic explanatory dataset    PCA analysis |
| Silt Percentage | Obtained from the same large-scale sampling campaign as species data in 2019. Silt percentage was considered the fraction of the sample with a grain size of < 63μm. Kriged to interpolate data for the entire Wadden sea, at 200m resolution. See [(Franken *et al.* 2025)](https://www.zotero.org/google-docs/?MyAcNd) for full methodology |  | In abiotic explanatory dataset    PCA analysis |
| Standard deviation Bathymetric Turnover | Bathymetric instability maps through the calculation of average annual bathymetric difference over the period of 1985-2020, at 20m resolution. | (Meijer *et al.* 2023a) | In abiotic explanatory dataset    PCA analysis |
| Mean Bathymetric Turnover |  |  | - |
| Slope | Slope map calculated in QGIS based off of the 2015-2020 bathymetry map, at 20m resolution. |  | In abiotic explanatory dataset    PCA analysis |
| Terrain Ruggedness Index | Terrain Ruggedness Index, at 20m resolution. TRI was extrapolated for the whole Dutch Wadden Sea from the TRI calculations for the Marsdiep tidal basin provided in Meijer *et al.* 2023a. |  | In abiotic explanatory dataset    PCA analysis |

# Appendix S3: Abiotic Variable Maps

| 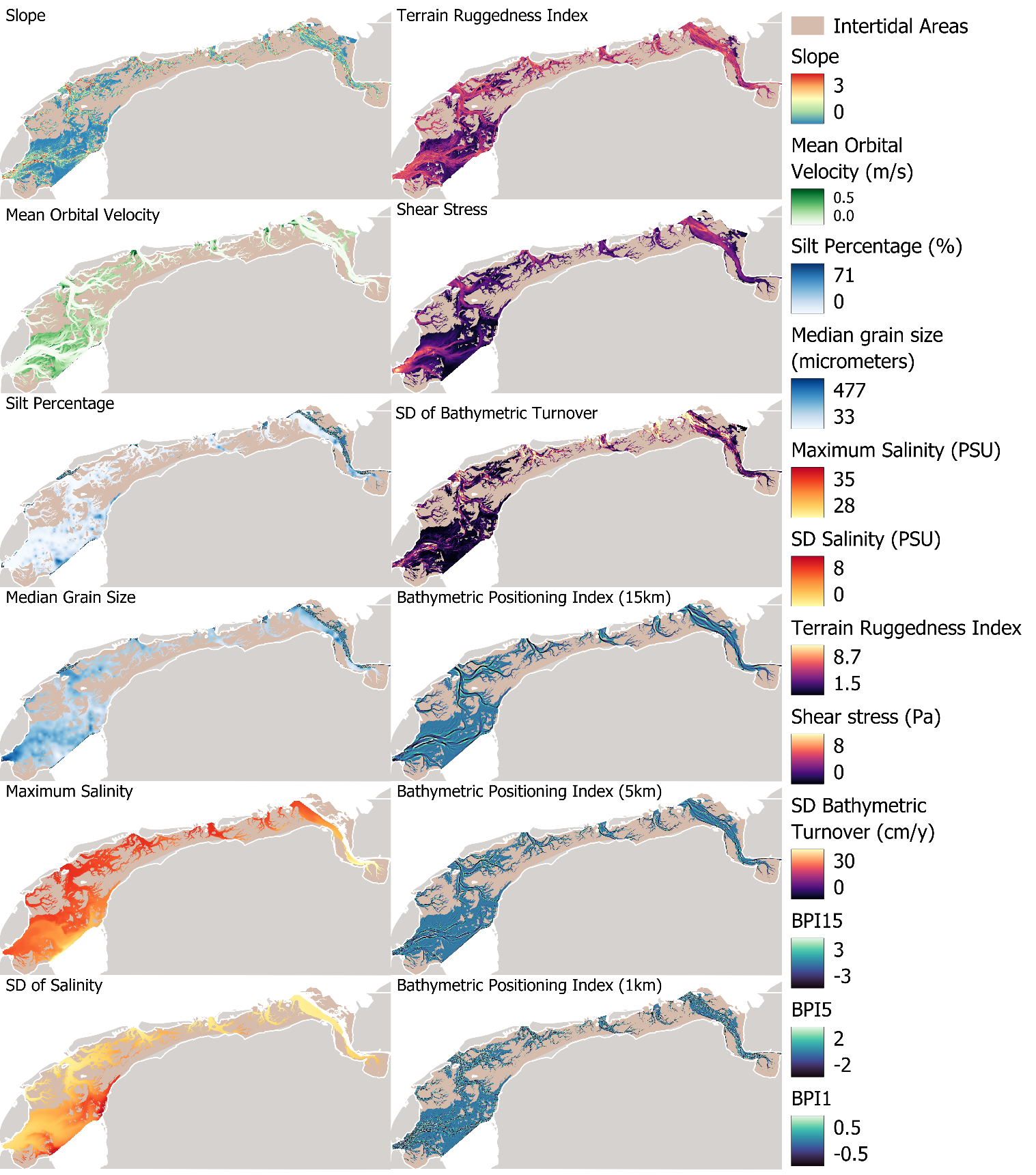 |
| --- |
| *Supplement 3:* Overview of the 12 environmental variables used in data analysis of this study, after removal of collinear variables using VIF analysis. |

# Appendix S4: Distribution of Abiotic Variables (related to Habitat Disturbance) across PC1 Disturbance Categories


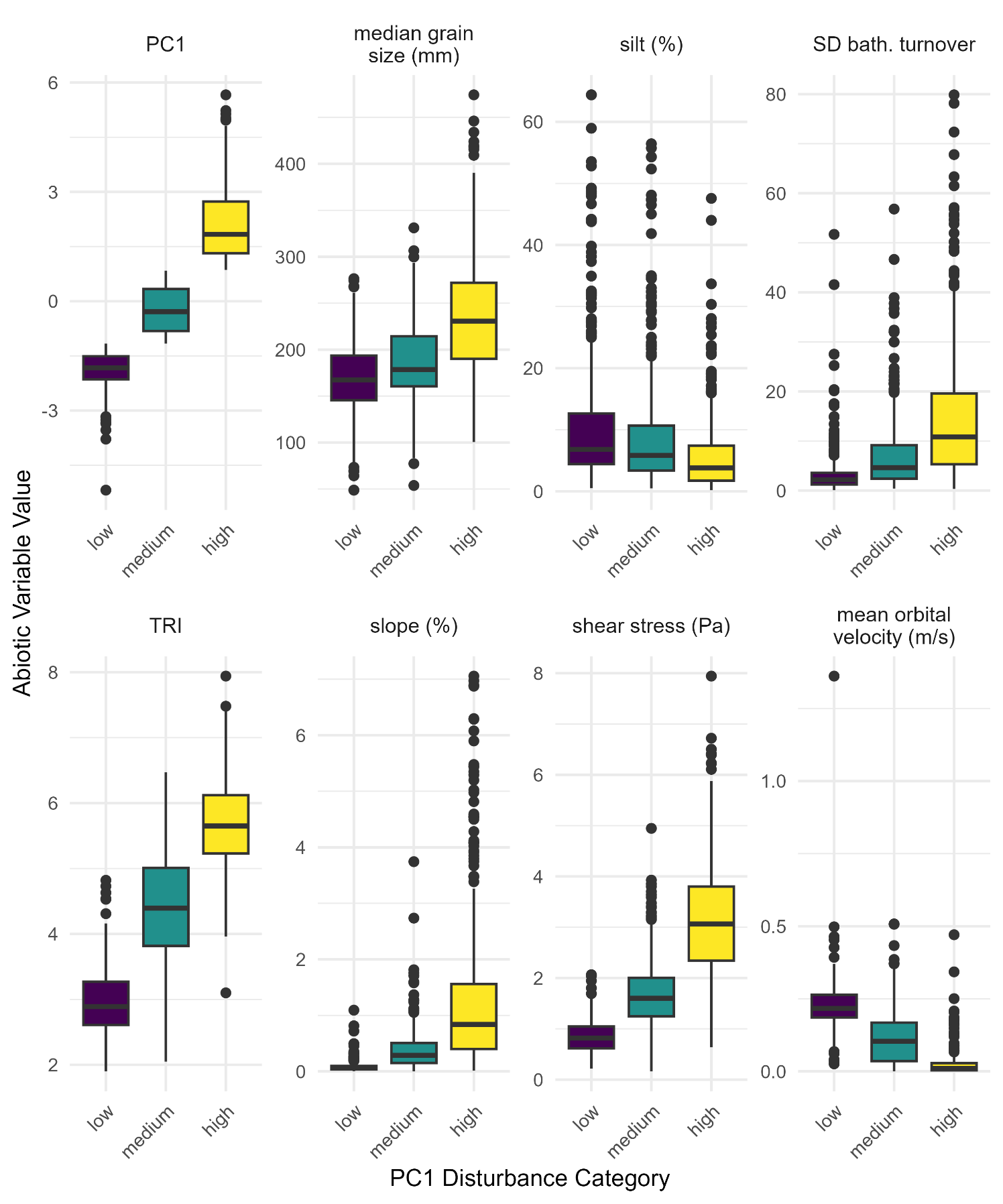


#

# Appendix S5: Overview of Weighted Average PC1 values


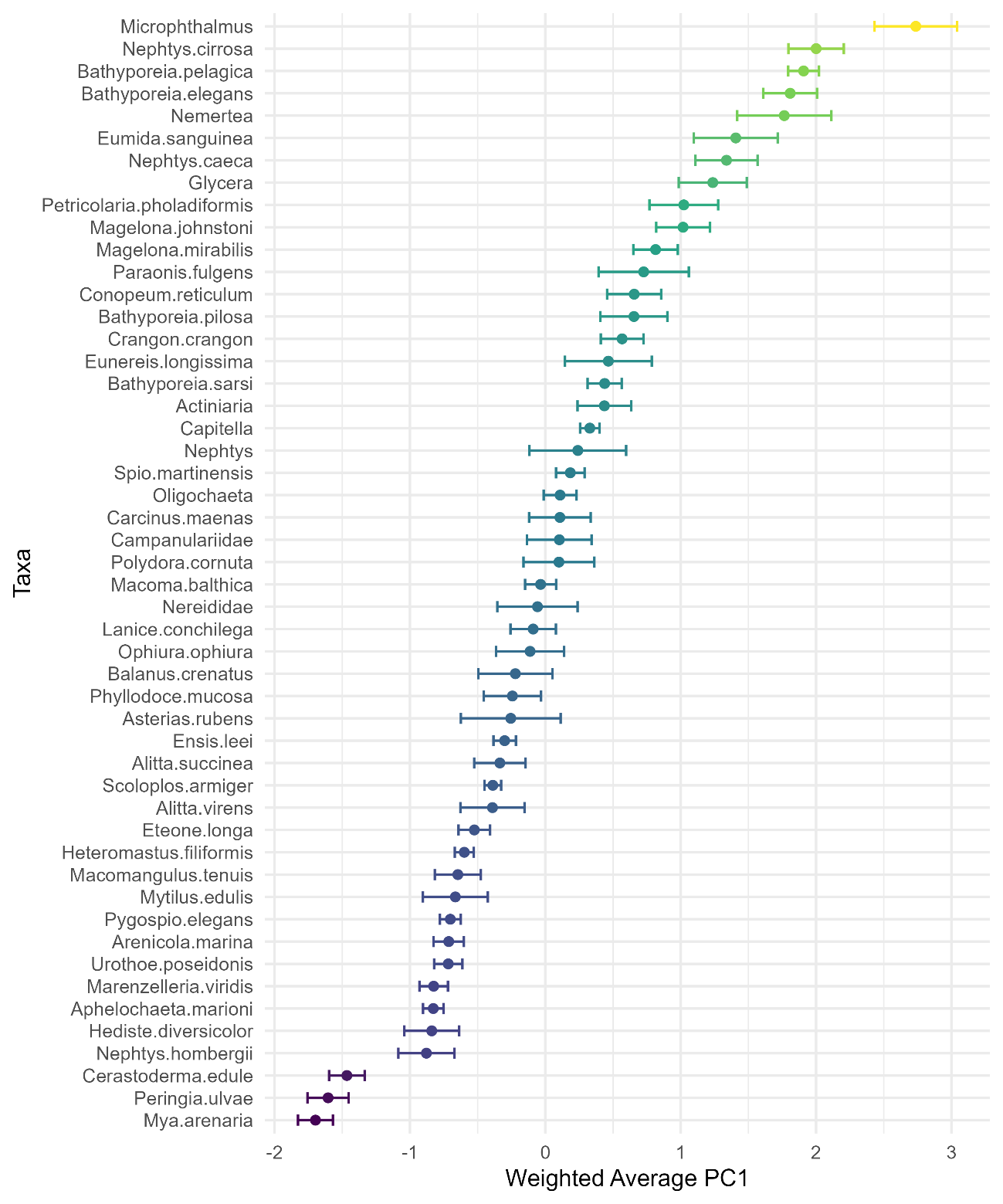


| *Supplement 5:* Weighted average of PC1 values per taxa, for the 50 taxa with the highest occupancy (i.e., the number of samples where the taxon was found). Error bars represent standard error. |
| --- |

#

# Appendix S6: DCA1 loadings of Trait Modalities

| **Supplement 3:** Table showing each macrozoobenthic trait modality and its associated DCA1 value. Modalities within traits are ordered from lowest DCA1 value to highest. | | |
| --- | --- | --- |
| **Trait** | **Modality** | **DCA1** |
| Age of sexual maturation (years) | >1 and ≤ 2 | -0.72 |
| Age of sexual maturation (years) | >2 and ≤ 5 | -0.27 |
| Age of sexual maturation (years) | ≤1 | 0.73 |
| Fecundity (reproductive output per reproductive event) | >50 and ≤ 500 | -0.61 |
| Fecundity (reproductive output per reproductive event) | >500 and ≤ 2.500 | -0.22 |
| Fecundity (reproductive output per reproductive event) | >2.500 and ≤ 10.000 | -0.15 |
| Fecundity (reproductive output per reproductive event) | >10.000 and ≤ 20.000 | -0.07 |
| Fecundity (reproductive output per reproductive event) | >20.000 and ≤ 100.000 | -0.02 |
| Fecundity (reproductive output per reproductive event) | >100.000 | 0.00 |
| Fecundity (reproductive output per reproductive event) | >=1 and ≤ 50 | 0.80 |
| Larval development location | Planktotrophic | -0.40 |
| Larval development location | Lecithotrophic | -0.23 |
| Larval development location | Benthic/Direct | 0.81 |
| Adult living habitat | Tube | -0.55 |
| Adult living habitat | Attached | -0.04 |
| Adult living habitat | Free-living | -0.02 |
| Adult living habitat | epi/endo-zoic/phytic | -0.01 |
| Adult living habitat | Crevice | 0.05 |
| Adult living habitat | Burrow | 0.45 |
| Offspring size (μm) | >100 and ≤ 500 | -0.68 |
| Offspring size (μm) | ≤ 100 | 0.00 |
| Offspring size (μm) | >1500 | 0.47 |
| Offspring size (μm) | >500 and ≤ 1500 | 0.62 |
| Offspring type | Egg | -0.65 |
| Offspring type | Larva | -0.08 |
| Offspring type | Juvenile | 0.86 |
| Reproductive mode | Broadcast | -0.53 |
| Reproductive mode | Benthic_Deposition | -0.20 |
| Reproductive mode | Asexual | -0.14 |
| Reproductive mode | Brooder | 0.67 |
| Reproductive frequency | Semelparous | -0.49 |
| Reproductive frequency | Annual 1x | -0.34 |
| Reproductive frequency | Continuous / ≥2x per year | 0.68 |
| Reproductive season | Spring | -0.27 |
| Reproductive season | Autumn | -0.15 |
| Reproductive season | Winter | -0.12 |
| Reproductive season | Summer | 0.53 |
| Adult movement | Swim/float | -0.26 |
| Adult movement | Sessile | -0.06 |
| Adult movement | Crawl/walk | -0.01 |
| Adult movement | Burrow/tube | 0.80 |
| Bioturbation type | Upward conveyor | -0.48 |
| Bioturbation type | Biodiffuser | -0.25 |
| Bioturbation type | Downward conveyor | -0.19 |
| Bioturbation type | Epifauna | 0.00 |
| Bioturbation type | Regenerator | 0.03 |
| Bioturbation type | Surficial modifier | 0.61 |
| Body size adults (mm) | >80 and ≤ 160 | -0.58 |
| Body size adults (mm) | >160 | -0.12 |
| Body size adults (mm) | >40 and ≤ 80 | -0.11 |
| Body size adults (mm) | >10 and ≤ 20 | -0.08 |
| Body size adults (mm) | >20 and ≤ 40 | 0.05 |
| Body size adults (mm) | ≤ 5 | 0.16 |
| Body size adults (mm) | >5 and ≤ 10 | 0.80 |
| Adult living depth (cm) | >25 | -0.53 |
| Adult living depth (cm) | >15 and ≤ 25 | -0.41 |
| Adult living depth (cm) | >8 and ≤ 15 | -0.06 |
| Adult living depth (cm) | >0 and ≤ 3 | 0.48 |
| Adult living depth (cm) | surface | 0.57 |
| Adult living depth (cm) | >3 and ≤ 8 | 0.59 |
| Feeding mode | Grazer | -0.06 |
| Feeding mode | Predator | -0.05 |
| Feeding mode | Deposit-feeder | -0.02 |
| Feeding mode | Suspension-feeder | 0.02 |
| Feeding mode | Opportunist/scavenger | 0.05 |
| Longevity (years) | >1 and ≤ 3 | -0.48 |
| Longevity (years) | >10 | -0.11 |
| Longevity (years) | >3 and ≤ 6 | -0.07 |
| Longevity (years) | >6 and ≤ 10 | 0.02 |
| Longevity (years) | ≤1 | 0.70 |
| Skeleton | Soft | -0.68 |
| Skeleton | Calcified | 0.03 |
| Skeleton | Chitin | 0.86 |

# 
